# The optimal approach for retrieving systematic reviews was achieved when searching MEDLINE and Epistemonikos in addition to reference checking: a methodological validation study

**DOI:** 10.1186/s12874-024-02384-2

**Published:** 2024-11-09

**Authors:** Lena Heinen, Käthe Goossen, Carole Lunny, Julian Hirt, Livia Puljak, Dawid Pieper

**Affiliations:** 1 Institute for Health Economics and Clinical Epidemiology (IGKE), School of Medicine, University of Cologne, Cologne, Germany; 2 Institute for Research in Operative Medicine (IFOM), Faculty of Health, School of Medicine, Witten/Herdecke University, Ostmerheimer Str. 200, 51109 Cologne, Germany; 3 Knowledge Translation Program, Unity Health Toronto and the Cochrane Hypertension Review Group, St Michael’s Hospital, University of British Columbia, Vancouver, Canada; 4 Department of Health, Eastern Switzerland University of Applied Sciences, St. Gallen, Switzerland; 5 Pragmatic Evidence Lab, Research Center for Clinical Neuroimmunology and Neuroscience Basel (RC2NB), University Hospital Basel and University of Basel, Basel, Switzerland; 6 Institute of Health and Nursing Science, Medical Faculty, Martin Luther University Halle-Wittenberg, Halle (Saale), Germany; 7 Center for Evidence-Based Medicine and Healthcare, Catholic University of Croatia, Zagreb, Croatia; 8 Faculty of Health Sciences Brandenburg, Brandenburg Medical School (Theodor Fontane), Institute for Health Services and Health System Research, Rüdersdorf, Germany; 9 Center for Health Services Research, Brandenburg Medical School (Theodor Fontane), Rüdersdorf, Germany

**Keywords:** Databases, Evidence synthesis, Geographical bias, Information specialist, Overview of review, Review methods, Search strategy, Systematic reviews, Umbrella review

## Abstract

**Background:**

Systematic reviews (SRs) are used to inform clinical practice guidelines and healthcare decision making by synthesising the results of primary studies. Efficiently retrieving as many relevant SRs as possible is challenging with a minimum number of databases, as there is currently no guidance on how to do this optimally. In a previous study, we determined which individual databases contain the most SRs, and which combination of databases retrieved the most SRs. In this study, we aimed to validate those previous results by using a different, larger, and more recent set of SRs.

**Methods:**

We obtained a set of 100 Overviews of Reviews that included a total of 2276 SRs. SR inclusion was assessed in MEDLINE, Embase, and Epistemonikos. The mean inclusion rates (% of included SRs) and corresponding 95% confidence intervals were calculated for each database individually, as well as for combinations of MEDLINE with each other database and reference checking. Features of SRs not identified by the best database combination were reviewed qualitatively.

**Results:**

Inclusion rates of SRs were similar in all three databases (mean inclusion rates in % with 95% confidence intervals: 94.3 [93.9–94.8] for MEDLINE, 94.4 [94.0-94.9] for Embase, and 94.4 [93.9–94.9] for Epistemonikos). Adding reference checking to MEDLINE increased the inclusion rate to 95.5 [95.1–96.0]. The best combination of two databases plus reference checking consisted of MEDLINE and Epistemonikos (98.1 [97.7–98.5]). Among the 44/2276 SRs not identified by this combination, 34 were published in journals from China, four were other journal publications, three were health agency reports, two were dissertations, and one was a preprint. When discounting the journal publications from China, the SR inclusion rate in the recommended combination (MEDLINE, Epistemonikos and reference checking) was even higher than in the previous study (99.6 vs. 99.2%).

**Conclusions:**

A combination of databases and reference checking was the best approach to searching for biomedical SRs. MEDLINE and Epistemonikos, complemented by checking the references of the included studies, was the most efficient and produced the highest recall. However, our results point to the presence of geographical bias, because some publications in journals from China were not identified.

**Study registration:**

10.17605/OSF.IO/R5EAS (Open Science Framework).

**Supplementary Information:**

The online version contains supplementary material available at 10.1186/s12874-024-02384-2.

## Background

Systematic reviews (SRs) are used to inform clinical practice guidelines, health policies, health technology assessments (HTAs), and public health mandates by synthesing the results of primary studies [1–3]. Searching multiple databases to retrieve SRs is arduous and time-consuming [4]. Database syntax and keywords are specific to each database, and translating a search strategy into multiple database interfaces and syntaxes is difficult, though automation tools are emerging that help with this task [5]. Differences in keywords between databases is also a significant challenge for translation. When importing records from multiple database searches, hundreds or thousands of irrelevant records need to be shifted through, making this a time-consuming effort [6]. Finally, access to some databases is available only on a subscription basis. Thus, it is worthwhile exploring how to efficiently retrieve as many relevant SRs as possible, with a minimum number of databases searched for maximum efficiency.

In an earlier study, we explored the question of which databases included the most systematic reviews and identified an optimal database combination for searching systematic reviews. The inclusion of a set of 1219 SRs published between 1982 and 2011 was assessed in MEDLINE, CINAHL, Embase, Epistemonikos, PsycINFO, and TRIP. In that study, > 99% of SRs were found by searching only two databases in combination – MEDLINE (via PubMed) [7] and Epistemonikos [8] in addition to reference checking (i.e. reviewing the reference lists of included studies manually or assisted by software tools) [9]. The other databases did not perform as well. Using our approach, the burden of searching for and retrieving SRs is streamlined and efficienct, without reducing the validity by missing relevant references.

The aim of the present follow-up study was to validate these previous results by using a different, larger, and more recent dataset of SRs and to compare these results with our earlier publication [10]. External validation against a set of SRs not included in the sample used to develop our recommended database combination allows for conclusions to be drawn about the generalisability of our findings [11]. The validation step is crucial because the performance measures of our database combination based on the initial dataset are likely to be overly optimistic.

## Methods

### Study design

A methodological study was conducted to analyse the rate of inclusion of SRs in three selected electronic databases, and the combination of two databases in addition to reference checking. The protocol for this study was registered prospectively with *Open Science Framework* (Center for Open Science, Charlottesville, VA) and can be accessed via 10.17605/OSF.IO/R5EAS. This study followed the methodology of our earlier study which is briefly outlined below [9]. Additional file 1 provides further methodological details.

### Data sources

We included a sample of 2276 SRs from another study [10]. The sample of SRs was extracted from 100 Overviews of SRs covering healthcare interventions for a wide range of medical conditions or public health problems.

### Description of electronic databases

We assessed whether SRs were indexed in three databases, namely MEDLINE (PubMed), Embase (Elsevier), and Epistemonikos. These three databases had the highest single-database inclusion rates in our earlier study. In contrast to the earlier study, we did not re-investigate the Cumulative Index to Nursing and Allied Health Literature (CINAHL), PsycINFO, and Trip databases, as these databases had low inclusion rates of 44.7%, 24.5% and 52.6% respectively. In addition, it did not appear necessary to re-investigate these databases because the overall inclusion rate in the recommended database combination was as high as 99.5% of SRs in the original dataset.

MEDLINE and Embase are databases covering the biomedicine and health literature. PubMed encompasses all of MEDLINE in addition to more recent publications. Epistemonikos is a meta-database that gathers SRs in the field of human health by searching the Cochrane Database of Systematic Reviews (CDSR), PubMed, Embase, CINAHL, PsycINFO, Latin American and Caribbean Health Sciences Literature, Database of Abstracts of Reviews of Effects (DARE), the Campbell Collaboration online library, Joanna Briggs Institute Database of Systematic Reviews and Implementation Reports, and Evidence for Policy and Practice Information and Co-ordinating Centre Evidence Library [12, 13]. CDSR was not included in the current study, because all Cochrane reviews are also included in MEDLINE [14].

### Data collection

All SRs were imported into Endnote (Version 20) and grouped into 100 subsets by topic. The topics were defined by the Overview the SRs originated from.

We searched for inclusion of the 2276 SRs in MEDLINE, Embase, and Epistemonikos in April and May 2022. To identify whether a SR was (i) included in an electronic database, (ii) found by reference checking, or (iii) included in a database combination (MEDLINE + Embase + reference checking, or MEDLINE + Epistemonikos + reference checking), a stepwise process was followed:

(A) MEDLINE was used as the reference database because it had the highest inclusion rate in our earlier study [9].

(B) A list of all SRs not included in MEDLINE was compiled. These SRs were called the ‘MEDLINE-non-included SRs’.

(C) A list of MEDLINE-non-included SR identified by reference checking was compiled. We used Scopus (www.scopus.com) to check the reference lists of the SRs retrieved from MEDLINE in the same subset by topic. The SRs found in the reference lists/bibliographies are henceforth called ‘SRs retrieved from reference checking’.

Finally, we constructed three sets of SRs: (i) SRs retrieved from MEDLINE + reference checking, (ii) SRs retrieved from MEDLINE + Embase + reference checking, and (iii) SRs retrieved from MEDLINE + Epistemonikos + reference checking. For each of these three sets, we calculated a mean inclusion rate (see statistical analysis). This was done to ascertain whether searching more than one database would identify additional SRs.

### Statistical analysis

For each of the 100 SR subsets by topic, we calculated:

(a) the mean inclusion rate (proportion of included SRs) separately for each database.

(b) the mean inclusion rate for MEDLINE in addition to reference checking.

(c) the mean inclusion rates for the combinations of MEDLINE, Embase, and reference checking, as well as MEDLINE, Epistemonikos, and reference checking.

The inclusion rates obtained in statistical analysis steps (a) to (c) were then aggregated for the entire dataset of 2276 SRs by calculating weighted mean inclusion rates and corresponding 95% CI. Weighting was based on the number of SRs included in each of the 100 subsets.

A threshold of 95% may be considered the limit below which the inclusion rate is inadequate, as suggested by other authors [15].

The mean inclusion rates of each database and each combination of databases investigated in this study were compared with the mean inclusion rates obtained in our earlier publication [9].

### Qualitative analysis of missed SRs

SRs that were not included by using a combined approach of searching MEDLINE, Epistemonikos, and reference checking (which was found to be the best combination) were analysed qualitatively. We looked at each SR individually and documented (i) the topics of these SRs, (ii) whether they were included in the other database analysed in the current study (i.e. Embase), (iii) whether they were included in any other database searched in the corresponding Overview (as described in the full text of the Overview), (iv) whether they could be located on websites (by a Google search for the SR title), (v) whether they were listed in a publisher’s database (we searched ScienceDirect, Wiley Online Library, SpringerLink, De Gruyter), or (vi) in Google Scholar (by typing the SR title into the search field).

### Changes compared to the registered protocol

The main analysis was conducted according to the protocol. However, we added a post-hoc analysis in which we excluded SRs from the main sample of SRs published in journals from China, which constituted the majority of missed SRs. 34 SRs published in journals from China were found. We then calculated our denominator as 2276 SRs minus 34 SRs published in journals from China equalling 2242 SRs. This additional analysis was included so that we could compare the results with and without SRs published in journals from China, and quantify their effect on the results.

## Results

### Study selection

A total of 2276 SRs published between 1990 and 2016 were included in 100 Overviews. Additional File 2 provides the topics for each of the 100 SR subsets, the number of SRs per topic, and bibliographic details of the source Overviews.

### SRs included in individual databases and database combinations

**Fig. 1:**
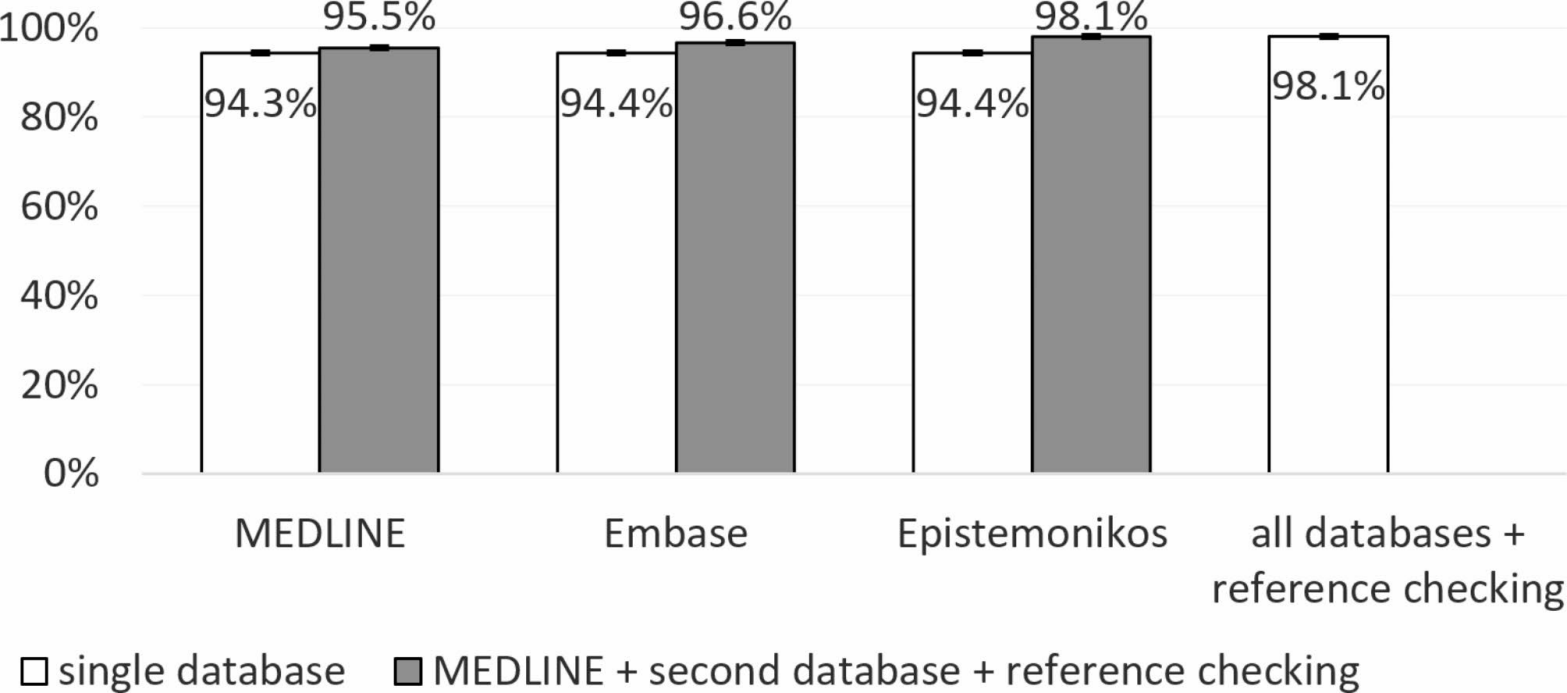
Inclusion rates [%] in individual databases and their combination with MEDLINE and reference checking

A large and similar proportion of SRs were included in MEDLINE (94.3%, *n* = 2147/2276), Embase (94.4%, *n* = 2149/2276), and Epistemonikos (94.4%, *n* = 2149/2276) (Table 1). 91% of SRs were included in both Embase and Epistemonikos (*n* = 2072/2276). Checking the references of MEDLINE-included SRs added a further 1.2% (*n* = 27/2276) to the SRs included in MEDLINE. In addition to MEDLINE and reference checking, Embase contributed an additional 1.1% (*n* = 25/2276) of SRs, Epistemonikos 2.6% (*n* = 58/2276). Combined inclusion rates are provided in Table 1 and illustrated in Fig. 1.

**Table 1:** Mean SR inclusion rate in individual databases and their combination with MEDLINE and reference checking

Database	Single database	Database + reference checking	MEDLINE + second database + reference checking
MEDLINE	94.33 [93.86–94.80]	95.52 [95.07–95.97]	
Embase	94.42 [93.95–94.89]	–	96.62 [96.18–97.06]
Epistemonikos	94.42 [93.94–94.90]	–	98.07 [97.67–98.46]

All values provided as percentages with 95% confidence interval

Figure 1 shows that MEDLINE, Epistemonikos, and reference checking together led to a SR inclusion rate of 98.1% (*n* = 2232/2276), as did a combination of all databases plus reference checking (*n* = 2233/2276, 98.1%). Therefore, there was no need to search Embase. A total of 1.9% (*n* = 43/2276) of SRs were not included in the “best combination” (MEDLINE, Epistemonikos, and reference checking).

None of the databases alone achieved a 95% threshold, but they did in combination (Table 1).

### Qualitative analysis of missed SRs

Forty-four SRs (*n* = 44/2276, 1.9%) were not included in the combination of MEDLINE, Epistemonikos, and reference checking. Most of these SRs (*n* = 34/44, 77.3%) were published in journals from China that are not currently indexed in any of the databases examined in this study (see Additional file 3). It was possible to identify 73.5% (*n* = 25/34) of these SRs using Google Scholar. With one exception [16], their topics relate to traditional Chinese medicine, such as acupuncture or Shenmai injection. When removing the SRs published in journals from China from the analysis, the best combination (MEDLINE, Epistemonikos, and reference checking) included 99.6% (*n* = 2232/2242) of the SRs.

Further analysis of the ten remaining missed SRs (Table 2) showed that two were included in CINAHL [17, 18], one in Embase [19], and none in Web of Science Core Collection. Thus, the addition of these three databases to the combination of MEDLINE, Epistemonikos, and reference checking would contribute only up to 0.1% (*n* = 3/2276) additional SRs to the overall set. A further three missed SRs [20–22] were reports by health technology agencies, none of which were available via the *International Network of Agencies for Health Technology Assessment* database. Two missed SRs [23, 24] were Master’s or doctoral theses. A SR by Baradan et al. from 2013 was in peer review at the time it was cited [25]. This unpublished version is not included in the best database combination, but the published version is included in all three databases investigated in this study [26].

**Table 2:** Missed SRs (i.e. not included in MEDLINE, Epistemonikos, and reference checking as well as not published in journals from China)

Reference	Title	Database inclusion	Google Scholar
Hemmingsson 2001 [19]	Influencing adherence to physical activity behavior change in obese adults	Embase	Y
Binns 2008 [17]	Does Tai Chi improve strength and balance in people with multiple sclerosis—the current literature	CINAHL	Y^e^
Conn 2003 [18]	Evidence-based interventions to increase physical activity among older adults	CINAHL	Y
Ashra 2015 [20]^a^	A systematic review and meta-analysis assessing the effectiveness of pragmatic lifestyle interventions for the prevention of type 2 diabetes mellitus in routine practice		Y^e^
Robson 2010 [22]^a^	A systematic review of the effectiveness of training and education for the protection of workers		Y^e^
Bernard 1994 [21]^a^	Évaluation des méthodes coélioscopiques en chirurgie digestive		Y^e^
Rudmik 2014 [22]	High volume sinonasal budesonide irrigations for chronic rhinosinusitis: an update on the safety and effectiveness		Y^e^
Huang 2009 [23]^b^	Effect of shenmai injection on the mortality rate and complications of patients with acute myocardial infarction: a meta-analysis		Y^e^
Qiong 2012 [24]^c^	[Salvia Injection for hypertensive hemorrhage: a meta-analysis]		–
Baradan 2013 [25]^d^	Teaching evidence-based medicine to undergraduate medical students: a systematic review and meta-analysis – under peer review		–

^a^ health agency report; ^b^ Master’s thesis; ^c^ dissertation; ^d^ preprint on an institutional website; ^e^ included in Google Scholar as a citation

Adding another bibliographic or HTA database thus had a negligible effect overall (*n* = 3/2276, 0.1%). Google Scholar had the potential to contribute 33 additional SRs (*n* = 33/2276, 1.4%), including 25 published in journals from China. However, 30 of these were included only as a “citation” with no link provided to the original source.

### Comparison of the results with our earlier publication

**Fig. 2:**
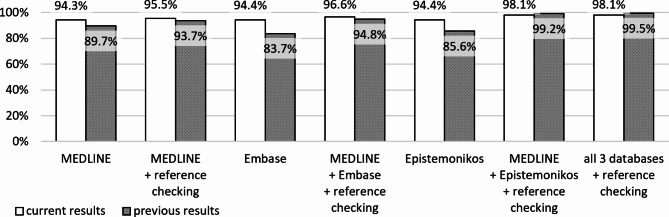
Inclusion rates for this study compared to our previous study

The SR inclusion rates in each individual database were higher compared to our previous study (Fig. 2). Whereas we previously found some differences between MEDLINE, Embase, and Epistemonikos (inclusion rates of 89.7%, 83.7%, and 85.6%, respectively), these were not found in the present study (94.3%, 94.4%, and 94.4%).

MEDLINE, Epistemonikos, and reference checking remained the best search combination for retrieving SRs. Compared to the previous study, the overall inclusion rate was slightly lower (99.2% for the previous vs. 98.1% for the current study). The difference may be attributed mainly to SRs published in journals from China (inclusion rate 99.6% when removing these from the analysis). The list of missed SRs in our earlier dataset did not contain any SR published in a journal from China.

## Discussion

The best approach to searching for and retrieving relevant SRs is to use a combination of the databases MEDLINE and Epistemonikos, complemented by reference checking, as this included over 95% of SRs on health-related topics. This combination should be used as a minimum requirement to guarantee adequate and efficient searching for biomedical SRs.

In this validation study, MEDLINE, Embase, and Epistemonikos obtained the same high inclusion rates. The inclusion rates were higher in each of the databases compared to our earlier study. This might be explained by the increasingly high overall number of journals indexed in these databases, which reduces earlier differences in journal coverage. According to the old version of the Cochrane Handbook for Systematic Reviews of Interventions published in 2011, MEDLINE and Embase included 5,200 and 4,800 journals, respectively [27]. Twelve years later (August 2023), MEDLINE and Embase included 5,280 and 8,501 journals, respectively [28, 29]. It is not surprising that Embase now has more indexed SRs than in our earlier study, and the single-database inclusion of Embase in this study equals that of MEDLINE. Since the Embase expansion in 2010, Embase should theoretically include all MEDLINE journals. It did not outperform MEDLINE, though, and the reason for this is unclear. If this study was validated again in the future using a new, even more recent dataset, we might expect Embase to outperform MEDLINE.

Epistemonikos automatically and regularly collects information from internet sources including both MEDLINE and Embase in addition to other databases. Thus, one could have expected that Epistemonikos would have yielded the highest SR inclusion rate, while in fact it was equal to Embase. A reason for this discrepancy may be that Epistemonikos is not a bibliographic database by definition [30]. Epistemonikos uses machine-based identification of records in bibliographic databases and other sources combined with manual validation [12]. This may lead to some misclassification errors. Another explanation is that Epistemonikos is a rather young source of evidence, and their methods (e.g. sources to search, when to search, SR classification methods) might have evolved over time.

Decision makers and investigators should consider carefully whether topic-specific or region-specific databases are likely to contain relevant SRs. For example, one study found that searching for studies on public health topics was very challenging (i.e. high likelihood of missing relevant evidence) and inefficient [31]. We also found that a small number of SRs which were published in Chinese-language journals were not indexed in our three databases. This suggests the presence of geographical bias. A likely explanation is our updated sample, as the number of Chinese-language journals is increasing steadily [32]. In addition, the topics for all but one of these missed SRs were related to traditional Chinese medicine. We therefore recommend searching Chinese language journals at least when looking for topics realeated to complementary and alternative medicine (as has been suggested for primary studies, e.g [33]). In order to limit the impact of geographical bias more effectively in the future, database providers should consider developing more inclusive strategies for coverage of international and non-English language resources. This could include collaborating with local databases and improving indexing of under-represented journals.

In our study, Google Scholar had a negligible effect in identifying additional references when used as a final-stage search resource after checking databases and references. Most articles accessible via Google Scholar were of the “citation” type, where no full text is available. Google Scholar is an inappropriate principal search platform as it is customized to the user’s computer device, and is not systematically objective [34]. Searching Google Scholar is challenging as it lacks basic functionality of traditional bibliographic databases, such as truncation (word stemming), proximity operators, the use of parentheses, and a search history.

### Comparison with previous findings

One previous study investigated the added value of different databases on different topics for retrieving SRs. Rathbone et al. (2016) evaluated the performance of major bibliographic databases to identify SRs on the topic of hypertension. They concluded that a search in all databases investigated (Cochrane library including CDSR, DARE, and HTA database, Embase, Epistemonikos, MEDLINE, PubMed Health and Trip) should be performed [35]. This is not in line with both our earlier study and our updated results. Possible reasons for this difference are related to both the dataset and the methods used. Rathbone used a dataset limited to hypertension, whereas our dataset topics varied. They evaluated the results of systematic searches in each database, whereas we limited our study to the aspect of database inclusion, and our results were not affected by the limitations of the search, i.e. incomplete recall, and screening errors. Further research is needed to assess whether adding databases might compensate for these limitations.

### Policy and guidance recommendations

Currently, there is no detailed guidance on how to search for SRs. Specific guidance for guideline developers, HTA representatives, information specialists, and Overview authors is needed to help identify the most relevant SRs on a given health topic. When developing such guidance, topic, setting and region-specific issues should be considered. Our results do not suffice to recommend that Embase replace MEDLINE when searching for SRs [36]. Currently, the advantage of its greater coverage of journals [28, 29] needs to be balanced with the disadvantage of its cost.

### Strengths and limitations

The major strength of our paper is that, together with our earlier study, it is the first large-scale investigation to determine the optimal databases and methods to retrieve SRs. Our current sample is much larger and more up-to-date than our earlier sample. This validation study confirms our previous results, and adds geographical bias as a new aspect to consider.

One of our study limitations is that we only relied on electronic databases for the inclusion of SRs. We did not hand-search all relevant resources for SRs, which has been considered by previous validation studies such as Davies et al. as a tool to obtain the gold standard dataset [37]. Davies et al. covered a specific population where it is easier to create a gold standard dataset via hand searching journals. Our study covered a wide range of populations, making hand searching impractical. We believe that in general, hand searching ought to be reconsidered as a method of validation in view of the high performance that Sampson et al. demonstrated for the relative recall method [38]. Another limitation is that the data source are SRs published in 2012–2016. The time lag between publication of the Overviews and publication of their included SRs, together with the time required to analyse and publish the subsequent meta-research, inevitably results in a somewhat older sample of SRs when generating datasets of SRs from Overviews of reviews. However, we suspect that an older sample would provide a more conservative estimate of database inclusion. Finally, the results may not apply specifically to grey literature reports due to limitations in the way we collected the included sample of SRs.

### Future research

Future studies might focus on several aspects. One would be to develop and validate effective and resource-efficient search strategies and search filters for SRs in the recommended database combination. Another would be to evaluate the generalisability of the results, for example by conducting similar studies in datasets specifically covering fields such as nursing, psychology, and allied health. Finally, future research might address the systematic development of guidance for identifying SRs.

## Conclusions

A combination of databases and reference checking was the best approach to searching for and retrieving relevant SRs. MEDLINE and Epistemonikos, complemented by checking the references of the included studies, was the most efficient and produced the highest recall. However, our results point to the presence of geographical bias, because some publications in journals from China were not identified. Special topics databases should be added depending on the research question of the SR, like Chinese journal databases for complementary and alternative medicine topics.

## Electronic supplementary material

Below is the link to the electronic supplementary material.

Supplementary Material 1

Supplementary Material 2

Supplementary Material 3

## Data Availability

Any data generated or analysed during this study that are not included in this published article or its supplementary information are available from the authors on request (Endnote files containing the SR dataset, Excel files containing the analysis).

## Abbreviations

CDSR: Cochrane Database of Systematic Reviews
CINAHL: Cumulative Index to Nursing and Allied Health Literature
DARE: Database of Abstracts of Reviews of Effects
HTA: Health Technology Assessment
SR: Systematic Review
