# Supplementary material for: The optimal approach for retrieving systematic reviews was achieved when searching MEDLINE and Epistemonikos in addition to reference checking: a methodological validation study
Source: BMC Med Res Methodol. 2024 Nov 9;24:271. doi: 10.1186/s12874-024-02384-2 (PMC11549827; doi:10.1186/s12874-024-02384-2)
Supplement: Supplementary file 2 — Supplementary Material 2 [file 12874_2024_2384_MOESM2_ESM.docx]

# Additional File 2. Topics of SRs, and characteristics of source Overviews

| **Topic** | **No. of SRs** | **Source Overview** | **DOI** | **PMID** | **No. of DBs searched** |
| --- | --- | --- | --- | --- | --- |
| Efficacy of Non-Pharmacological Interventions to Prevent and Treat Delirium in Older Patients | 26 | Abraha (2015) [1] | 10.1371/journal.pone.0123090 | 26062023 | 5 |
| Adverse Event Reporting for Proton Pump Inhibitor Therapy | 25 | Abramowitz (2016) [2] | 10.1177/0194599816648298 | 27188706 | 4 |
| Comparative effectiveness of new oral anticoagulants and standard thromboprophylaxis in patients having total hip or knee replacement | 6 | Adam (2013) [3] | 10.7326/0003-4819-159-4-201308200-00008 | 24026260 | 3 |
| Effectiveness of brief interventions in primary health care settings to decrease alcohol consumption by adult non-dependent drinkers | 7 | Alvarez-Bueno (2015) [4] | 10.1016/j.ypmed.2014.12.010 | 25514547 | 7 |
| The effect of clinical interventions on hospital readmissions | 97 | Benbassat (2013) [5] | 10.1186/2045-4015-2-1 | 23343012 | 7 |
| Preventing Child and Adolescent Anxiety Disorders | 3 | Bennett (2015) [6] | 10.1002/da.22400 | 26282454 | 7 |
| Treating child and adolescent anxiety effectively | 22 | Bennett (2016) [7] | 10.1016/j.cpr.2016.09.006 | 27744168 | 7 |
| Exercise for adults with fibromyalgia | 9 | Bidonde (2014) [8] | 10.2174/1573403x10666140914155304 | 25229499 | 6 |
| Public Health Interventions for Aedes Control in the Time of Zikavirus | 13 | Bouzid (2016) [9] | 10.1371/journal.pntd.0005176 | 27926934 | 2 |
| What works in community-based interventions promoting physical activity and healthy eating? | 18 | Brand (2014) [10] | 10.3390/ijerph110605866 | 24886756 | 7 |
| Endometriosis | 17 | Brown (2014) [11] | 10.1002/14651858.CD009590.pub2 | 24610050 | 2 |
| Dental and skeletal effects of palatal expansion techniques | 12 | Bucci (2016)[12] | 10.1111/joor.12393 | 27004835 | 8 |
| Chinese herbal medicine and depression | 6 | Butler (2013) [13] | 10.1155/2013/739716 | 23476701 | 10 |
| Pharmacological interventions for smoking cessation | 12 | Cahill (2013) [14] | 10.1002/14651858.CD009329.pub2 | 23728690 | 1 |
| Does Syndrome Differentiation Matter? | 5 | Cao (2012) [15] | 10.1089/acu.2011.0846 | 24761164 | 3 |
| Acute asthma treatment in childhood | 27 | Castro-Rodriguez (2015) [16] | 10.3109/02770903.2015.1033725 | 26377055 | 5 |
| The management of acute bronchiolitis in children | 20 | Castro-Rodriguez (2015) [17] | 10.1016/j.prrv.2014.11.004 | 25636596 | 5 |
| Exercise improves depressive symptoms in older adults | 3 | Catalan-Matamoros (2016) [18] | 10.1016/j.psychres.2016.07.028 | 27494042 | 5 |
| Environmental components of childhood obesity prevention interventions | 62 | Cauchi (2016) [19] | 10.1111/obr.12441 | 27432025 | 9 |
| Enhanced recovery programmes in colorectal surgery | 10 | Chambers (2014) [20] | 10.1136/bmjopen-2014-005014 | 24879828 | 3 |
| Malignancy risk of anti-tumor necrosis factor alpha blockers | 42 | Chen (2016) [21] | 10.1007/s10067-015-3115-7 | 26573205 | 5 |
| The effectiveness of reminders in improving healthcare professional behavior | 35 | Cheung (2012) [22] | 10.1186/2046-4053-1-36 | 22898173 | na |
| Physical rehabilitation interventions for adult patients during critical illness | 5 | Connolly (2016) [23] | 10.1136/thoraxjnl-2015-208273 | 27220357 | 6 |
| Interventions for Adolescent Substance Abuse | 46 | Das (2016) [24] | 10.1016/j.jadohealth.2016.06.021 | 27664597 | 4 |
| Effects of pay for performance in health care | 22 | Eijkenaar (2013) [25] | 10.1016/j.healthpol.2013.01.008 | 23380190 | 5 |
| Effectiveness and cost-effectiveness of ehealth interventions in somatic diseases | 31 | Elbert (2014) [26] | 10.2196/jmir.2790 | 24739471 | 6 |
| Mediterranean diet and type 2 diabetes | 8 | Esposito (2015) [27] | 10.1136/bmjopen-2015-008222 | 26260349 | 6 |
| Psychosocial treatment for attention-deficit/hyperactivity disorder | 12 | Fabiano (2015) [28] | 10.1007/s10567-015-0178-6 | 25691358 | 2 |
| The use of systemic antimicrobials for the treatment of periodontitis | 9 | Faggion Jr. (2014) [29] | 10.1038/sj.bdj.2014.909 | 25342355 | 2 |
| Treatment of acute gastroenteritis in children of interventions commonly used in developed countries | 4 | Freedman (2013) [30] | 10.1002/ebch.1932 | 23877938 | 8 |
| Patient compliance with medication protocols used in highly active antiretroviral therapy | 9 | Fu (2015) [31] | http://dx.doi.org/10.1016/j.ijnss.2015.02.005 | 602902543 | 10 |
| Incretin-based medications for type 2 diabetes | 84 | Gamble (2015) [32] | 10.1111/dom.12465 | 25772666 | 7 |
| Standardised mindfulness-based interventions in healthcare | 23 | Gotink (2015) [33] | 10.1371/journal.pone.0124344 | 25881019 | 6 |
| What do we know about mobile applications for diabetes self-management? | 11 | Hood (2016) [34] | 10.1007/s10865-016-9765-3 | 27412774 | 1 |
| Clinical impact of lifestyle interventions for the prevention of diabetes | 19 | Howells (2016) [35] | 10.1136/bmjopen-2016-013806 | 28003299 | 10 |
| Efficacy of pharmacotherapy and psychotherapy for adult psychiatric disorders | 61 | Huhn (2014) [36] | 10.1001/jamapsychiatry.2014.112 | 24789675 | 3 |
| Laparoscopic versus open appendectomy in patients with suspected appendicitis | 9 | Jaschinski (2015) [37] | 10.1186/s12876-015-0277-3 | 25884671 | 5 |
| The Effects of Clinical Decision Support Systems on Medication Safety | 20 | Jia (2016) [38] | 10.1371/journal.pone.0167683 | 27977697 | 5 |
| Effectiveness of music therapy | 21 | Kamioka (2014) [39] | 10.2147/PPA.S61340 | 24876768 | 12 |
| Dietary supplements for benign prostatic hyperplasia | 6 | Kim (2012) [40] | 10.1016/j.maturitas.2012.07.007 | 22883375 | 7 |
| Effects of home telemonitoring interventions on patients with chronic heart failure | 14 | Kitsiou (2015) [41] | 10.2196/jmir.4174 | 25768664 | 6 |
| The effectiveness of massage therapy for the treatment of nonspecific low back pain | 9 | Kumar (2013) [42] | 10.2147/IJGM.S50243 | 24043951 | 17 |
| Measures used to treat contrast-induced nephropathy | 7 | Kwok (2013) [43] | 10.1259/bjr.20120272 | 23239696 | 5 |
| Evidence-based dentistry | 3 | Lang (2014) [44] | 10.1016/j.prosdent.2013.12.001 | 24589122 | 4 |
| Complementary and Alternative Therapies in the Treatment of the Fibromyalgia Syndrome | 25 | Lauche (2015) [45] | 10.1155/2015/610615 | 26246841 | 5 |
| Interventions to delay functional decline in people with dementia | 23 | Laver (2016) [46] | 10.1136/bmjopen-2015-010767 | 27121704 | 5 |
| Acupuncture for low back pain | 16 | Liu (2015) [47] | 10.1155/2015/328196 | 25821485 | 9 |
| Anterior cruciate ligament reconstruction rehabilitation | 5 | Lobb (2012) [48] | 10.1016/j.ptsp.2012.05.001 | 23068905 | 10 |
| Shenmai injection for healthcare | 20 | Lu (2014) [49] | 10.1155/2014/840650 | 24669229 | 6 |
| Behavioral Interventions for Preventing Sexually Transmitted Infections and Unintended Pregnancies | 55 | Macaya Pascual (2016) [50] | 10.1016/j.ad.2015.10.010 | 26801866 | 8 |
| Laparoscopic surgery for colorectal cancer | 27 | Martel (2012) [51] | 10.1186/2046-4053-1-14 | 22588035 | 8 |
| The impact of self-monitoring in chronic illness on healthcare utilisation | 17 | McBain (2015) [52] | 10.1186/s12913-015-1221-5 | 26684011 | 7 |
| Yoga as a therapeutic intervention for adults with acute and chronic health conditions | 26 | McCall (2013) [53] | 10.1155/2013/945895 | 23762174 | 10 |
| The impact of telehealthcare on the quality and safety of care | 80 | McLean (2013) [54] | 10.1371/journal.pone.0071238 | 23977001 | 9 |
| Effectiveness of health care professionals using handheld computers | 5 | Mickan (2013) [55] | 10.2196/jmir.2530 | 24165786 | 8 |
| Non-prescription (OTC) oral analgesics for acute pain | 10 | Moore (2015) [56] | 10.1002/14651858.CD010794.pub2 | 26544675 | 1 |
| Changing physician behavior | 14 | Mostofian (2015) [57] | 25880152 |  | 3 |
| Interventions for treating pain and disability in adults with complex regional pain syndrome | 19 | O'Connell (2013) [58] | 10.1002/14651858.CD009416.pub2 | 23633371 | 7 |
| Physical Activity in Community Dwelling Older People | 40 | Olanrewaju (2016) [59] | 10.1371/journal.pone.0168614 | 27997604 | 9 |
| Influenza vaccination | 12 | Osterhus (2014) [60] | http://dx.doi.org/10.1007/s10096-014-2236-2 | 601628859 | 1 |
| Self-Management Support Interventions for Stroke Survivors | 13 | Parke (2015) [61] | 10.1371/journal.pone.0131448 | 26204266 | 8 |
| Pharmacological strategies for the prevention of acute kidney injury following cardiac surgery | 7 | Patel (2014) [62] | 10.2174/1381612820666140325113422 | 24669971 | 2 |
| Effectiveness of two interventions in preventing traffic accidents | 8 | Porchia (2014) [63] | 10.7416/ai.2014.1959 | 24452185 | 13 |
| Exercise prescription for hospitalized people with chronic obstructive pulmonary disease and comorbidities | 58 | Reid (2012) [64] | 10.2147/COPD.S29750 | 22665994 | 9 |
| The management of urinary incontinence and promotion of continence using conservative behavioural approaches in older people in care homes | 5 | Roe (2015) [65] | 10.1111/jan.12613 | 25615885 | 5 |
| Effectiveness of clinical pharmacy services (2000-2010) | 49 | Rotta (2015) [66] | 10.1007/s11096-015-0137-9 | 26001356 | 1 |
| Medical Therapies for Adult Chronic Sinusitis | 25 | Rudmik (2015) [67] | 10.1001/jama.2015.7544 | 26325561 | 4 |
| Interventions to improve safe and effective medicines use by consumers | 76 | Ryan (2014) [68] | 10.1002/14651858.CD007768.pub3 | 24777444 | 2 |
| Efficacy of inter-dental mechanical plaque control in managing gingivitis | 6 | Salzer (2015) [69] | 10.1111/jcpe.12363 | 25581718 | 5 |
| Assessing the State of Knowledge Regarding the Effectiveness of Interventions to Contain Pandemic Influenza Transmission and Narrative Synthesis | 17 | Saunders-Hastings (2016) [70] | 10.1371/journal.pone.0168262 | 27977760 | 7 |
| Self-management of oral anticoagulation | 8 | Siebenhofer (2014) [71] | 10.3238/arztebl.2014.0083 | 24622604 | 1 |
| Pediatric oral health | 37 | Smail-Faugeron (2014) [72] | 10.1186/1472-6831-14-35 | 24716532 | 1 |
| The effect of Tai Chi on health outcomes | 107 | Solloway (2016) [73] | 10.1186/s13643-016-0300-y | 27460789 | 10 |
| Improving diet and physical activity to reduce population prevalence of overweight and obesity | 83 | Stephens (2014) [74] | 10.1016/j.ypmed.2014.02.008 | 24534460 | 5 |
| What Works to Prevent Falls in Community-Dwelling Older Adults? | 16 | Stubbs (2015) [75] | 10.2522/ptj.20140461 | 25655877 | 11 |
| What works to prevent falls in older adults dwelling in long term care facilities and hospitals? | 10 | Stubbs (2015) [76] | 10.1016/j.maturitas.2015.03.026 | 25935294 | 11 |
| Efficacy, tolerability, and safety of cannabinoids for chemotherapy-induced nausea and vomiting | 6 | Tafelski (2016) [77] | 10.1007/s00482-015-0092-3 | 26787227 | 5 |
| Self-directed interventions to promote weight loss | 20 | Tang (2014) [78] | 10.2196/jmir.2857 | 24554464 | 7 |
| Vitamin D and multiple health outcomes | 97 | Theodoratou (2014) [79] | 10.1136/bmj.g2035 | 24690624 | 2 |
| Interventions to reduce emergency department utilisation | 23 | Van den Heede (2016) [80] | 10.1016/j.healthpol.2016.10.002 | 27855964 | 5 |
| Efficacy of homecare regimens for mechanical plaque removal in managing gingivitis | 10 | Van der Weijden (2015) [81] | 10.1111/jcpe.12359 | 25597787 | 5 |
| Can Chemical Mouthwash Agents Achieve Plaque/Gingivitis Control? | 14 | Van der Weijden (2015) [82] | 10.1016/j.cden.2015.06.002 | 26427569 | 5 |
| Essential Occupational Safety and Health Interventions for Low- and Middle-income Countries | 23 | Verbeek (2013) [83] | 10.1016/j.shaw.2013.04.004 | 23961329 | 2 |
| Interventions to enhance work participation of workers with a chronic disease | 9 | Vooijs (2015) [84] | 10.1136/oemed-2015-103062 | 26408509 | 7 |
| Effects of exercise on anxiety and depression disorders | 35 | Wegner (2014) [85] | 10.2174/1871527313666140612102841 | 24923346 | 7 |
| Interventions for bronchiectasis | 21 | Welsh (2015) [86] | 10.1002/14651858.CD010337.pub2 | 26171905 | 1 |
| Are non-steroidal anti-inflammatory drugs effective for the management of neck pain and associated disorders, whiplash-associated disorders, or non-specific low back pain? | 8 | Wong (2016) [87] | 10.1007/s00586-015-3891-4 | 25827308 | 6 |
| Dressings for treating foot ulcers in people with diabetes | 13 | Wu (2015) [88] | 10.1002/14651858.CD010471.pub2 | 26171906 | 4 |
| Effectiveness of acupuncture and related therapies for palliative care of cancer | 23 | Wu (2015) [89] | 10.1038/srep16776 | 26608664 | 7 |
| Is It Necessary To Place Prophylactically an Abdominal Drain To Prevent Surgical Site Infection in Abdominal Operations? | 15 | Wu (2016) [90] | 10.1089/sur.2016.082 | 27513842 | 6 |
| Chinese herbal medicine for the treatment of primary hypertension | 12 | Xinke (2016) [91] | 10.1186/s13643-016-0353-y | 27760557 | 5 |
| Effects of Exercise on Bone Status in Female Subjects, from Young Girls to Postmenopausal Women | 12 | Xu (2016) [92] | 10.1007/s40279-016-0494-0 | 26856338 | 6 |
| Interventions for tic disorders | 22 | Yang (2016) [93] | 10.1016/j.neubiorev.2015.12.013 | 26751711 | 9 |
| Integrated multidisciplinary care for the management of chronic conditions in adults | 7 | Yeung (2016)[94] | 10.1111/hae.13010 | 27348400 | 4 |
| What are the effects of teaching evidence-based health care (EBHC)? | 16 | Young (2014) [95] | 10.1371/journal.pone.0086706 | 24489771 | 9 |
| Adverse drug reactions due to drug-drug interactions with proton pump inhibitors | 20 | Yucel (2016) [96] | 10.1517/14740338.2016.1128413 | 26635063 | 8 |
| Acupuncture for chronic nonspecific low back pain | 17 | Zeng (2015) [97] | http://dx.doi.org/10.1016/j.eujim.2014.11.001 | 603595133 | 7 |
| Ginkgo biloba Extracts for Mild Cognitive Impairment and Dementia | 10 | Zhang (2016) [98] | 10.3389/fnagi.2016.00276 | 27999539 | 6 |
| Traditional Chinese Patent Medicine for Acute Ischemic Stroke | 9 | Zhang (2016) [99] | 10.1097/MD.0000000000002986 | 27015174 | 9 |
| Danhong Injection for Unstable Angina | 5 | Zhang (2015) [100] | 10.1155/2015/358028 | 26539221 | 9 |

1. Abraha I, Trotta F, Rimland JM, Cruz-Jentoft A, Lozano-Montoya I, Soiza RL, et al. Efficacy of Non-Pharmacological Interventions to Prevent and Treat Delirium in Older Patients: A Systematic Overview. The SENATOR project ONTOP Series. PLoS ONE. 2015;10(6):e0123090. doi: <https://dx.doi.org/10.1371/journal.pone.0123090>. PubMed PMID: 26062023.

2. Abramowitz J, Thakkar P, Isa A, Truong A, Park C, Rosenfeld RM. Adverse Event Reporting for Proton Pump Inhibitor Therapy: An Overview of Systematic Reviews. Otolaryngol Head Neck Surg. 2016;155(4):547-54. doi: <https://dx.doi.org/10.1177/0194599816648298>. PubMed PMID: 27188706.

3. Adam SS, McDuffie JR, Lachiewicz PF, Ortel TL, Williams JW, Jr. Comparative effectiveness of new oral anticoagulants and standard thromboprophylaxis in patients having total hip or knee replacement: a systematic review. Ann Intern Med. 2013;159(4):275-84. doi: <https://dx.doi.org/10.7326/0003-4819-159-4-201308200-00008>. PubMed PMID: 24026260.

4. Alvarez-Bueno C, Rodriguez-Martin B, Garcia-Ortiz L, Gomez-Marcos MA, Martinez-Vizcaino V. Effectiveness of brief interventions in primary health care settings to decrease alcohol consumption by adult non-dependent drinkers: a systematic review of systematic reviews. Prev Med. 2015;76 Suppl:S33-8. doi: <https://dx.doi.org/10.1016/j.ypmed.2014.12.010>. PubMed PMID: 25514547.

5. Benbassat J, Taragin MI. The effect of clinical interventions on hospital readmissions: a meta-review of published meta-analyses. Isr J Health Policy Res. 2013;2(1):1. doi: <https://dx.doi.org/10.1186/2045-4015-2-1>. PubMed PMID: 23343012.

6. Bennett K, Manassis K, Duda S, Bagnell A, Bernstein GA, Garland EJ, et al. Preventing Child and Adolescent Anxiety Disorders: Overview of Systematic Reviews. Depress Anxiety. 2015;32(12):909-18. doi: <https://dx.doi.org/10.1002/da.22400>. PubMed PMID: 26282454.

7. Bennett K, Manassis K, Duda S, Bagnell A, Bernstein GA, Garland EJ, et al. Treating child and adolescent anxiety effectively: Overview of systematic reviews. Clin Psychol Rev. 2016;50:80-94. doi: <https://dx.doi.org/10.1016/j.cpr.2016.09.006>. PubMed PMID: 27744168.

8. Bidonde J, Busch AJ, Bath B, Milosavljevic S. Exercise for adults with fibromyalgia: an umbrella systematic review with synthesis of best evidence. Curr Rheumatol Rev. 2014;10(1):45-79. doi: <https://dx.doi.org/10.2174/1573403x10666140914155304>. PubMed PMID: 25229499.

9. Bouzid M, Brainard J, Hooper L, Hunter PR. Public Health Interventions for Aedes Control in the Time of Zikavirus- A Meta-Review on Effectiveness of Vector Control Strategies. PLoS Negl Trop Dis. 2016;10(12):e0005176. doi: <https://dx.doi.org/10.1371/journal.pntd.0005176>. PubMed PMID: 27926934.

10. Brand T, Pischke CR, Steenbock B, Schoenbach J, Poettgen S, Samkange-Zeeb F, et al. What works in community-based interventions promoting physical activity and healthy eating? A review of reviews. Int J Environ Res Public Health. 2014;11(6):5866-88. doi: <https://dx.doi.org/10.3390/ijerph110605866>. PubMed PMID: 24886756.

11. Brown J, Farquhar C. Endometriosis: an overview of Cochrane Reviews. Cochrane Database Syst Rev. 2014;(3):CD009590. doi: <https://dx.doi.org/10.1002/14651858.CD009590.pub2>. PubMed PMID: 24610050.

12. Bucci R, D'Anto V, Rongo R, Valletta R, Martina R, Michelotti A. Dental and skeletal effects of palatal expansion techniques: a systematic review of the current evidence from systematic reviews and meta-analyses. J Oral Rehabil. 2016;43(7):543-64. doi: <https://dx.doi.org/10.1111/joor.12393>. PubMed PMID: 27004835.

13. Butler L, Pilkington K. Chinese herbal medicine and depression: the research evidence. Evid Based Complement Alternat Med. 2013;2013:739716. doi: <https://dx.doi.org/10.1155/2013/739716>. PubMed PMID: 23476701.

14. Cahill K, Stevens S, Perera R, Lancaster T. Pharmacological interventions for smoking cessation: an overview and network meta-analysis. Cochrane Database Syst Rev. 2013;(5):CD009329. doi: <https://dx.doi.org/10.1002/14651858.CD009329.pub2>. PubMed PMID: 23728690.

15. Cao H, Bourchier S, Liu J. Does Syndrome Differentiation Matter? A Meta-Analysis of Randomized Controlled Trials in Cochrane Reviews of Acupuncture. Med Acupunct. 2012;24(2):68-76. doi: <https://dx.doi.org/10.1089/acu.2011.0846>. PubMed PMID: 24761164.

16. Castro-Rodriguez JA, G JR, C ER-M. Principal findings of systematic reviews of acute asthma treatment in childhood.[Erratum appears in J Asthma. 2016;53(3):339; PMID: 26377055]. J Asthma. 2015;52(10):1038-45. doi: <https://dx.doi.org/10.3109/02770903.2015.1033725>. PubMed PMID: 26303207.

17. Castro-Rodriguez JA, Rodriguez-Martinez CE, Sossa-Briceno MP. Principal findings of systematic reviews for the management of acute bronchiolitis in children. Paediatr Respir Rev. 2015;16(4):267-75. doi: <https://dx.doi.org/10.1016/j.prrv.2014.11.004>. PubMed PMID: 25636596.

18. Catalan-Matamoros D, Gomez-Conesa A, Stubbs B, Vancampfort D. Exercise improves depressive symptoms in older adults: An umbrella review of systematic reviews and meta-analyses. Psychiatry Res. 2016;244:202-9. doi: <https://dx.doi.org/10.1016/j.psychres.2016.07.028>. PubMed PMID: 27494042.

19. Cauchi D, Glonti K, Petticrew M, Knai C. Environmental components of childhood obesity prevention interventions: an overview of systematic reviews. Obes Rev. 2016;17(11):1116-30. doi: <https://dx.doi.org/10.1111/obr.12441>. PubMed PMID: 27432025.

20. Chambers D, Paton F, Wilson P, Eastwood A, Craig D, Fox D, et al. An overview and methodological assessment of systematic reviews and meta-analyses of enhanced recovery programmes in colorectal surgery. BMJ Open. 2014;4(5):e005014. doi: <https://dx.doi.org/10.1136/bmjopen-2014-005014>. PubMed PMID: 24879828.

21. Chen Y, Sun J, Yang Y, Huang Y, Liu G. Malignancy risk of anti-tumor necrosis factor alpha blockers: an overview of systematic reviews and meta-analyses. Clin Rheumatol. 2016;35(1):1-18. doi: <https://dx.doi.org/10.1007/s10067-015-3115-7>. PubMed PMID: 26573205.

22. Cheung A, Weir M, Mayhew A, Kozloff N, Brown K, Grimshaw J. Overview of systematic reviews of the effectiveness of reminders in improving healthcare professional behavior. Syst. 2012;1:36. doi: <https://dx.doi.org/10.1186/2046-4053-1-36>. PubMed PMID: 22898173.

23. Connolly B, O'Neill B, Salisbury L, Blackwood B, Enhanced Recovery After Critical Illness Programme G. Physical rehabilitation interventions for adult patients during critical illness: an overview of systematic reviews. Thorax. 2016;71(10):881-90. doi: <https://dx.doi.org/10.1136/thoraxjnl-2015-208273>. PubMed PMID: 27220357.

24. Das JK, Salam RA, Arshad A, Finkelstein Y, Bhutta ZA. Interventions for Adolescent Substance Abuse: An Overview of Systematic Reviews. J Adolesc Health. 2016;59(4S):S61-S75. doi: <https://dx.doi.org/10.1016/j.jadohealth.2016.06.021>. PubMed PMID: 27664597.

25. Eijkenaar F, Emmert M, Scheppach M, Schoffski O. Effects of pay for performance in health care: a systematic review of systematic reviews. Health Policy. 2013;110(2-3):115-30. doi: <https://dx.doi.org/10.1016/j.healthpol.2013.01.008>. PubMed PMID: 23380190.

26. Elbert NJ, van Os-Medendorp H, van Renselaar W, Ekeland AG, Hakkaart-van Roijen L, Raat H, et al. Effectiveness and cost-effectiveness of ehealth interventions in somatic diseases: a systematic review of systematic reviews and meta-analyses. J Med Internet Res. 2014;16(4):e110. doi: <https://dx.doi.org/10.2196/jmir.2790>. PubMed PMID: 24739471.

27. Esposito K, Maiorino MI, Bellastella G, Chiodini P, Panagiotakos D, Giugliano D. A journey into a Mediterranean diet and type 2 diabetes: a systematic review with meta-analyses. BMJ Open. 2015;5(8):e008222. doi: <https://dx.doi.org/10.1136/bmjopen-2015-008222>. PubMed PMID: 26260349.

28. Fabiano GA, Schatz NK, Aloe AM, Chacko A, Chronis-Tuscano A. A systematic review of meta-analyses of psychosocial treatment for attention-deficit/hyperactivity disorder. Clin Child Fam Psychol Rev. 2015;18(1):77-97. doi: <https://dx.doi.org/10.1007/s10567-015-0178-6>. PubMed PMID: 25691358; PubMed Central PMCID: PMCNIHMS665323.

29. Faggion CM, Jr., Cullinan MP, Atieh M, Wasiak J. An overview of systematic reviews of the use of systemic antimicrobials for the treatment of periodontitis. Br Dent J. 2014;217(8):443-51. doi: <https://dx.doi.org/10.1038/sj.bdj.2014.909>. PubMed PMID: 25342355.

30. Freedman SB, Ali S, Oleszczuk M, Gouin S, Hartling L. Treatment of acute gastroenteritis in children: an overview of systematic reviews of interventions commonly used in developed countries. Evid Based Child Health. 2013;8(4):1123-37. doi: <https://dx.doi.org/10.1002/ebch.1932>. PubMed PMID: 23877938.

31. Fu L, Hu Y, Lu HZ. Overviews of reviews on patient compliance with medication protocols used in highly active antiretroviral therapy. International Journal of Nursing Sciences. 2015;2(1):61-5. doi: <http://dx.doi.org/10.1016/j.ijnss.2015.02.005>. PubMed PMID: 602902543.

32. Gamble JM, Clarke A, Myers KJ, Agnew MD, Hatch K, Snow MM, et al. Incretin-based medications for type 2 diabetes: an overview of reviews. Diabetes Obes Metab. 2015;17(7):649-58. doi: <https://dx.doi.org/10.1111/dom.12465>. PubMed PMID: 25772666.

33. Gotink RA, Chu P, Busschbach JJ, Benson H, Fricchione GL, Hunink MG. Standardised mindfulness-based interventions in healthcare: an overview of systematic reviews and meta-analyses of RCTs. PLoS ONE. 2015;10(4):e0124344. doi: <https://dx.doi.org/10.1371/journal.pone.0124344>. PubMed PMID: 25881019.

34. Hood M, Wilson R, Corsica J, Bradley L, Chirinos D, Vivo A. What do we know about mobile applications for diabetes self-management? A review of reviews. J Behav Med. 2016;39(6):981-94. doi: <https://dx.doi.org/10.1007/s10865-016-9765-3>. PubMed PMID: 27412774.

35. Howells L, Musaddaq B, McKay AJ, Majeed A. Clinical impact of lifestyle interventions for the prevention of diabetes: an overview of systematic reviews. BMJ Open. 2016;6(12):e013806. doi: <https://dx.doi.org/10.1136/bmjopen-2016-013806>. PubMed PMID: 28003299.

36. Huhn M, Tardy M, Spineli LM, Kissling W, Forstl H, Pitschel-Walz G, et al. Efficacy of pharmacotherapy and psychotherapy for adult psychiatric disorders: a systematic overview of meta-analyses. JAMA Psychiatry. 2014;71(6):706-15. doi: <https://dx.doi.org/10.1001/jamapsychiatry.2014.112>. PubMed PMID: 24789675.

37. Jaschinski T, Mosch C, Eikermann M, Neugebauer EA. Laparoscopic versus open appendectomy in patients with suspected appendicitis: a systematic review of meta-analyses of randomised controlled trials. BMC Gastroenterol. 2015;15:48. doi: <https://dx.doi.org/10.1186/s12876-015-0277-3>. PubMed PMID: 25884671.

38. Jia P, Zhang L, Chen J, Zhao P, Zhang M. The Effects of Clinical Decision Support Systems on Medication Safety: An Overview. PLoS ONE. 2016;11(12):e0167683. doi: <https://dx.doi.org/10.1371/journal.pone.0167683>. PubMed PMID: 27977697.

39. Kamioka H, Tsutani K, Yamada M, Park H, Okuizumi H, Tsuruoka K, et al. Effectiveness of music therapy: a summary of systematic reviews based on randomized controlled trials of music interventions. Patient preference and adherence. 2014;8:727. Epub 20140516. doi: <https://dx.doi.org/10.2147/PPA.S61340>. PubMed PMID: 24876768; PubMed Central PMCID: PMCPMC4036702.

40. Kim TH, Lim HJ, Kim MS, Lee MS. Dietary supplements for benign prostatic hyperplasia: an overview of systematic reviews. Maturitas. 2012;73(3):180-5. doi: <https://dx.doi.org/10.1016/j.maturitas.2012.07.007>. PubMed PMID: 22883375.

41. Kitsiou S, Pare G, Jaana M. Effects of home telemonitoring interventions on patients with chronic heart failure: an overview of systematic reviews. J Med Internet Res. 2015;17(3):e63. doi: <https://dx.doi.org/10.2196/jmir.4174>. PubMed PMID: 25768664.

42. Kumar S, Beaton K, Hughes T. The effectiveness of massage therapy for the treatment of nonspecific low back pain: a systematic review of systematic reviews. Int J Gen Med. 2013;6:733-41. doi: <https://dx.doi.org/10.2147/IJGM.S50243>. PubMed PMID: 24043951.

43. Kwok CS, Pang CL, Yeong JK, Loke YK. Measures used to treat contrast-induced nephropathy: overview of reviews. Br J Radiol. 2013;86(1021):20120272. doi: <https://dx.doi.org/10.1259/bjr.20120272>. PubMed PMID: 23239696.

44. Lang LA, Teich ST. A critical appraisal of evidence-based dentistry: the best available evidence. J Prosthet Dent. 2014;111(6):485-92. doi: <https://dx.doi.org/10.1016/j.prosdent.2013.12.001>. PubMed PMID: 24589122.

45. Lauche R, Cramer H, Hauser W, Dobos G, Langhorst J. A Systematic Overview of Reviews for Complementary and Alternative Therapies in the Treatment of the Fibromyalgia Syndrome. Evid Based Complement Alternat Med. 2015;2015:610615. doi: <https://dx.doi.org/10.1155/2015/610615>. PubMed PMID: 26246841.

46. Laver K, Dyer S, Whitehead C, Clemson L, Crotty M. Interventions to delay functional decline in people with dementia: a systematic review of systematic reviews. BMJ Open. 2016;6(4):e010767. doi: <https://dx.doi.org/10.1136/bmjopen-2015-010767>. PubMed PMID: 27121704.

47. Liu L, Skinner M, McDonough S, Mabire L, Baxter GD. Acupuncture for low back pain: an overview of systematic reviews. Evid Based Complement Alternat Med. 2015;2015:328196. doi: <https://dx.doi.org/10.1155/2015/328196>. PubMed PMID: 25821485.

48. Lobb R, Tumilty S, Claydon LS. A review of systematic reviews on anterior cruciate ligament reconstruction rehabilitation. Phys Ther Sport. 2012;13(4):270-8. doi: <https://dx.doi.org/10.1016/j.ptsp.2012.05.001>. PubMed PMID: 23068905.

49. Lu LY, Zheng GQ, Wang Y. An overview of systematic reviews of shenmai injection for healthcare. Evid Based Complement Alternat Med. 2014;2014:840650. doi: <https://dx.doi.org/10.1155/2014/840650>. PubMed PMID: 24669229.

50. Macaya Pascual A, Ferreres Riera JR, Campoy Sanchez A. Behavioral Interventions for Preventing Sexually Transmitted Infections and Unintended Pregnancies: An Overview of Systematic Reviews. Actas Dermosifiliogr. 2016;107(4):301-17. doi: <https://dx.doi.org/10.1016/j.ad.2015.10.010>. PubMed PMID: 26801866.

51. Martel G, Duhaime S, Barkun JS, Boushey RP, Ramsay CR, Fergusson DA. The quality of research synthesis in surgery: the case of laparoscopic surgery for colorectal cancer. Syst. 2012;1:14. doi: <https://dx.doi.org/10.1186/2046-4053-1-14>. PubMed PMID: 22588035.

52. McBain H, Shipley M, Newman S. The impact of self-monitoring in chronic illness on healthcare utilisation: a systematic review of reviews. BMC Health Serv Res. 2015;15:565. doi: <https://dx.doi.org/10.1186/s12913-015-1221-5>. PubMed PMID: 26684011.

53. McCall MC, Ward A, Roberts NW, Heneghan C. Overview of systematic reviews: yoga as a therapeutic intervention for adults with acute and chronic health conditions. Evid Based Complement Alternat Med. 2013;2013:945895. doi: <https://dx.doi.org/10.1155/2013/945895>. PubMed PMID: 23762174.

54. McLean S, Sheikh A, Cresswell K, Nurmatov U, Mukherjee M, Hemmi A, et al. The impact of telehealthcare on the quality and safety of care: a systematic overview. PLoS ONE. 2013;8(8):e71238. doi: <https://dx.doi.org/10.1371/journal.pone.0071238>. PubMed PMID: 23977001.

55. Mickan S, Tilson JK, Atherton H, Roberts NW, Heneghan C. Evidence of effectiveness of health care professionals using handheld computers: a scoping review of systematic reviews. J Med Internet Res. 2013;15(10):e212. doi: <https://dx.doi.org/10.2196/jmir.2530>. PubMed PMID: 24165786.

56. Moore RA, Wiffen PJ, Derry S, Maguire T, Roy YM, Tyrrell L. Non-prescription (OTC) oral analgesics for acute pain - an overview of Cochrane reviews. Cochrane Database Syst Rev. 2015;(11):CD010794. doi: <https://dx.doi.org/10.1002/14651858.CD010794.pub2>. PubMed PMID: 26544675.

57. Mostofian F, Ruban C, Simunovic N, Bhandari M. Changing physician behavior: what works? Am J Manag Care. 2015;21(1):75-84. PubMed PMID: 25880152.

58. O'Connell NE, Wand BM, McAuley J, Marston L, Moseley GL. Interventions for treating pain and disability in adults with complex regional pain syndrome. Cochrane Database Syst Rev. 2013;(4):CD009416. doi: <https://dx.doi.org/10.1002/14651858.CD009416.pub2>. PubMed PMID: 23633371.

59. Olanrewaju O, Kelly S, Cowan A, Brayne C, Lafortune L. Physical Activity in Community Dwelling Older People: A Systematic Review of Reviews of Interventions and Context. PLoS ONE. 2016;11(12):e0168614. doi: <https://dx.doi.org/10.1371/journal.pone.0168614>. PubMed PMID: 27997604.

60. Osterhus SF. Influenza vaccination: a summary of Cochrane Reviews. European Journal of Clinical Microbiology and Infectious Diseases. 2014;34(2):205-13. doi: <http://dx.doi.org/10.1007/s10096-014-2236-2>. PubMed PMID: 601628859.

61. Parke HL, Epiphaniou E, Pearce G, Taylor SJ, Sheikh A, Griffiths CJ, et al. Self-Management Support Interventions for Stroke Survivors: A Systematic Meta-Review. PLoS ONE. 2015;10(7):e0131448. doi: <https://dx.doi.org/10.1371/journal.pone.0131448>. PubMed PMID: 26204266.

62. Patel NN, Angelini GD. Pharmacological strategies for the prevention of acute kidney injury following cardiac surgery: an overview of systematic reviews. Curr Pharm Des. 2014;20(34):5484-8. doi: <https://dx.doi.org/10.2174/1381612820666140325113422>. PubMed PMID: 24669971.

63. Porchia BR, Baldasseroni A, Dellisanti C, Lorini C, Bonaccorsi G. Effectiveness of two interventions in preventing traffic accidents: a systematic review. Ann Ig. 2014;26(1):63-75. doi: <https://dx.doi.org/10.7416/ai.2014.1959>. PubMed PMID: 24452185.

64. Reid WD, Yamabayashi C, Goodridge D, Chung F, Hunt MA, Marciniuk DD, et al. Exercise prescription for hospitalized people with chronic obstructive pulmonary disease and comorbidities: a synthesis of systematic reviews. Int J Chron Obstruct Pulmon Dis. 2012;7:297-320. doi: <https://dx.doi.org/10.2147/COPD.S29750>. PubMed PMID: 22665994.

65. Roe B, Flanagan L, Maden M. Systematic review of systematic reviews for the management of urinary incontinence and promotion of continence using conservative behavioural approaches in older people in care homes. J Adv Nurs. 2015;71(7):1464-83. doi: <https://dx.doi.org/10.1111/jan.12613>. PubMed PMID: 25615885.

66. Rotta I, Salgado TM, Silva ML, Correr CJ, Fernandez-Llimos F. Effectiveness of clinical pharmacy services: an overview of systematic reviews (2000-2010). Int J Clin Pharm. 2015;37(5):687-97. doi: <https://dx.doi.org/10.1007/s11096-015-0137-9>. PubMed PMID: 26001356.

67. Rudmik L, Soler ZM. Medical Therapies for Adult Chronic Sinusitis: A Systematic Review. Jama. 2015;314(9):926-39. doi: <https://dx.doi.org/10.1001/jama.2015.7544>. PubMed PMID: 26325561.

68. Ryan R, Santesso N, Lowe D, Hill S, Grimshaw J, Prictor M, et al. Interventions to improve safe and effective medicines use by consumers: an overview of systematic reviews. Cochrane Database Syst Rev. 2014;(4):CD007768. doi: <https://dx.doi.org/10.1002/14651858.CD007768.pub3>. PubMed PMID: 24777444.

69. Salzer S, Slot DE, Van der Weijden FA, Dorfer CE. Efficacy of inter-dental mechanical plaque control in managing gingivitis--a meta-review. J Clin Periodontol. 2015;42 Suppl 16:S92-105. doi: <https://dx.doi.org/10.1111/jcpe.12363>. PubMed PMID: 25581718.

70. Saunders-Hastings P, Reisman J, Krewski D. Assessing the State of Knowledge Regarding the Effectiveness of Interventions to Contain Pandemic Influenza Transmission: A Systematic Review and Narrative Synthesis. PLoS ONE. 2016;11(12):e0168262. doi: <https://dx.doi.org/10.1371/journal.pone.0168262>. PubMed PMID: 27977760.

71. Siebenhofer A, Jeitler K, Horvath K, Habacher W, Schmidt L, Semlitsch T. Self-management of oral anticoagulation. Dtsch. 2014;111(6):83-91. doi: <https://dx.doi.org/10.3238/arztebl.2014.0083>. PubMed PMID: 24622604.

72. Smail-Faugeron V, Fron-Chabouis H, Courson F. Methodological quality and implications for practice of systematic Cochrane reviews in pediatric oral health: a critical assessment. BMC Oral Health. 2014;14:35. doi: <https://dx.doi.org/10.1186/1472-6831-14-35>. PubMed PMID: 24716532.

73. Solloway MR, Taylor SL, Shekelle PG, Miake-Lye IM, Beroes JM, Shanman RM, et al. An evidence map of the effect of Tai Chi on health outcomes. Syst. 2016;5(1):126. doi: <https://dx.doi.org/10.1186/s13643-016-0300-y>. PubMed PMID: 27460789.

74. Stephens SK, Cobiac LJ, Veerman JL. Improving diet and physical activity to reduce population prevalence of overweight and obesity: an overview of current evidence. Prev Med. 2014;62:167-78. doi: <https://dx.doi.org/10.1016/j.ypmed.2014.02.008>. PubMed PMID: 24534460.

75. Stubbs B, Brefka S, Denkinger MD. What Works to Prevent Falls in Community-Dwelling Older Adults? Umbrella Review of Meta-analyses of Randomized Controlled Trials. Phys Ther. 2015;95(8):1095-110. doi: <https://dx.doi.org/10.2522/ptj.20140461>. PubMed PMID: 25655877.

76. Stubbs B, Denkinger MD, Brefka S, Dallmeier D. What works to prevent falls in older adults dwelling in long term care facilities and hospitals? An umbrella review of meta-analyses of randomised controlled trials. Maturitas. 2015;81(3):335-42. doi: <https://dx.doi.org/10.1016/j.maturitas.2015.03.026>. PubMed PMID: 25935294.

77. Tafelski S, Hauser W, Schafer M. Efficacy, tolerability, and safety of cannabinoids for chemotherapy-induced nausea and vomiting--a systematic review of systematic reviews. Schmerz. 2016;30(1):14-24. doi: <https://dx.doi.org/10.1007/s00482-015-0092-3>. PubMed PMID: 26787227.

78. Tang J, Abraham C, Greaves C, Yates T. Self-directed interventions to promote weight loss: a systematic review of reviews. J Med Internet Res. 2014;16(2):e58. Epub 20140219. doi: <https://dx.doi.org/10.2196/jmir.2857>. PubMed PMID: 24554464; PubMed Central PMCID: PMCPMC3961624.

79. Theodoratou E, Tzoulaki I, Zgaga L, Ioannidis JP. Vitamin D and multiple health outcomes: umbrella review of systematic reviews and meta-analyses of observational studies and randomised trials. Bmj. 2014;348:g2035. doi: <https://dx.doi.org/10.1136/bmj.g2035>. PubMed PMID: 24690624.

80. Van den Heede K, Van de Voorde C. Interventions to reduce emergency department utilisation: A review of reviews. Health Policy. 2016;120(12):1337-49. doi: <https://dx.doi.org/10.1016/j.healthpol.2016.10.002>. PubMed PMID: 27855964.

81. Van der Weijden FA, Slot DE. Efficacy of homecare regimens for mechanical plaque removal in managing gingivitis a meta review. J Clin Periodontol. 2015;42 Suppl 16:S77-91. doi: <https://dx.doi.org/10.1111/jcpe.12359>. PubMed PMID: 25597787.

82. Van der Weijden FA, Van der Sluijs E, Ciancio SG, Slot DE. Can Chemical Mouthwash Agents Achieve Plaque/Gingivitis Control? Dent Clin North Am. 2015;59(4):799-829. doi: <https://dx.doi.org/10.1016/j.cden.2015.06.002>. PubMed PMID: 26427569.

83. Verbeek J, Ivanov I. Essential Occupational Safety and Health Interventions for Low- and Middle-income Countries: An Overview of the Evidence. Saf Health Work. 2013;4(2):77-83. doi: <https://dx.doi.org/10.1016/j.shaw.2013.04.004>. PubMed PMID: 23961329.

84. Vooijs M, Leensen MC, Hoving JL, Wind H, Frings-Dresen MH. Interventions to enhance work participation of workers with a chronic disease: a systematic review of reviews. Occup Environ Med. 2015;72(11):820-6. doi: <https://dx.doi.org/10.1136/oemed-2015-103062>. PubMed PMID: 26408509.

85. Wegner M, Helmich I, Machado S, Nardi AE, Arias-Carrion O, Budde H. Effects of exercise on anxiety and depression disorders: review of meta- analyses and neurobiological mechanisms. CNS Neurol Disord Drug Targets. 2014;13(6):1002-14. doi: <https://dx.doi.org/10.2174/1871527313666140612102841>. PubMed PMID: 24923346.

86. Welsh EJ, Evans DJ, Fowler SJ, Spencer S. Interventions for bronchiectasis: an overview of Cochrane systematic reviews. Cochrane Database Syst Rev. 2015;(7):CD010337. doi: <https://dx.doi.org/10.1002/14651858.CD010337.pub2>. PubMed PMID: 26171905.

87. Wong JJ, Cote P, Ameis A, Varatharajan S, Varatharajan T, Shearer HM, et al. Are non-steroidal anti-inflammatory drugs effective for the management of neck pain and associated disorders, whiplash-associated disorders, or non-specific low back pain? A systematic review of systematic reviews by the Ontario Protocol for Traffic Injury Management (OPTIMa) Collaboration. Eur Spine J. 2016;25(1):34-61. doi: <https://dx.doi.org/10.1007/s00586-015-3891-4>. PubMed PMID: 25827308.

88. Wu L, Norman G, Dumville JC, O'Meara S, Bell-Syer SE. Dressings for treating foot ulcers in people with diabetes: an overview of systematic reviews. Cochrane Database Syst Rev. 2015;(7):CD010471. doi: <https://dx.doi.org/10.1002/14651858.CD010471.pub2>. PubMed PMID: 26171906.

89. Wu X, Chung VC, Hui EP, Ziea ET, Ng BF, Ho RS, et al. Effectiveness of acupuncture and related therapies for palliative care of cancer: overview of systematic reviews. Sci. 2015;5:16776. doi: <https://dx.doi.org/10.1038/srep16776>. PubMed PMID: 26608664.

90. Wu X, Tian W, Kubilay NZ, Ren J, Li J. Is It Necessary To Place Prophylactically an Abdominal Drain To Prevent Surgical Site Infection in Abdominal Operations? A Systematic Meta-Review. Surg Infect (Larchmt). 2016;17(6):730-8. doi: <https://dx.doi.org/10.1089/sur.2016.082>. PubMed PMID: 27513842.

91. Xinke Z, Yingdong L, Mingxia F, Kai L, Kaibing C, Yuqing L, et al. Chinese herbal medicine for the treatment of primary hypertension: a methodology overview of systematic reviews. Syst. 2016;5(1):180. doi: <https://dx.doi.org/10.1186/s13643-016-0353-y>. PubMed PMID: 27760557.

92. Xu J, Lombardi G, Jiao W, Banfi G. Effects of Exercise on Bone Status in Female Subjects, from Young Girls to Postmenopausal Women: An Overview of Systematic Reviews and Meta-Analyses. Sports Med. 2016;46(8):1165-82. doi: <https://dx.doi.org/10.1007/s40279-016-0494-0>. PubMed PMID: 26856338.

93. Yang C, Hao Z, Zhu C, Guo Q, Mu D, Zhang L. Interventions for tic disorders: An overview of systematic reviews and meta analyses. Neurosci Biobehav Rev. 2016;63:239-55. doi: <https://dx.doi.org/10.1016/j.neubiorev.2015.12.013>. PubMed PMID: 26751711.

94. Yeung CH, Santesso N, Zeraatkar D, Wang A, Pai M, Sholzberg M, et al. Integrated multidisciplinary care for the management of chronic conditions in adults: an overview of reviews and an example of using indirect evidence to inform clinical practice recommendations in the field of rare diseases. Haemophilia. 2016;22 Suppl 3:41-50. doi: <https://dx.doi.org/10.1111/hae.13010>. PubMed PMID: 27348400.

95. Young T, Rohwer A, Volmink J, Clarke M. What are the effects of teaching evidence-based health care (EBHC)? Overview of systematic reviews. PLoS ONE. 2014;9(1):e86706. doi: <https://dx.doi.org/10.1371/journal.pone.0086706>. PubMed PMID: 24489771.

96. Yucel E, Sancar M, Yucel A, Okuyan B. Adverse drug reactions due to drug-drug interactions with proton pump inhibitors: assessment of systematic reviews with AMSTAR method. Expert Opin Drug Saf. 2016;15(2):223-36. doi: <https://dx.doi.org/10.1517/14740338.2016.1128413>. PubMed PMID: 26635063.

97. Zeng Y, Chung JWY. Acupuncture for chronic nonspecific low back pain: An overview of systematic reviews. European Journal of Integrative Medicine. 2015;7(2):94-107. doi: <http://dx.doi.org/10.1016/j.eujim.2014.11.001>. PubMed PMID: 603595133.

98. Zhang HF, Huang LB, Zhong YB, Zhou QH, Wang HL, Zheng GQ, et al. An Overview of Systematic Reviews of Ginkgo biloba Extracts for Mild Cognitive Impairment and Dementia. Front Aging Neurosci. 2016;8:276. doi: <https://dx.doi.org/10.3389/fnagi.2016.00276>. PubMed PMID: 27999539.

99. Zhang X, Liu XT, Kang DY. Traditional Chinese Patent Medicine for Acute Ischemic Stroke: An Overview of Systematic Reviews Based on the GRADE Approach. Medicine (Baltimore). 2016;95(12):e2986. doi: <https://dx.doi.org/10.1097/MD.0000000000002986>. PubMed PMID: 27015174.

100. Zhang X, Wang H, Chang Y, Wang Y, Lei X, Fu S, et al. An Overview of Meta-Analyses of Danhong Injection for Unstable Angina. Evid Based Complement Alternat Med. 2015;2015:358028. doi: <https://dx.doi.org/10.1155/2015/358028>. PubMed PMID: 26539221.
