# Supplementary material for: The optimal approach for retrieving systematic reviews was achieved when searching MEDLINE and Epistemonikos in addition to reference checking: a methodological validation study
Source: BMC Med Res Methodol. 2024 Nov 9;24:271. doi: 10.1186/s12874-024-02384-2 (PMC11549827; doi:10.1186/s12874-024-02384-2)
Supplement: Supplementary file 3 — Supplementary Material 3 [file 12874_2024_2384_MOESM3_ESM.docx]

# Additional file 3. Systematic reviews published in Chinese journals.

| **Reference** | **Title** | **Journal** |
| --- | --- | --- |
| Qin 2010 [1]^a^ | Systematic review of randomized controlled trials of Xiaoyao powder in treatment of depression | Journal of Traditional Chinese Medicine |
| Wu 2013 [2]^a^ | Acupuncture for lumbar disc herniation: a systematic review | Journal of Guangxi Medical University |
| Li 2010 [3]^a^ | Clinical randomized controlled trials on treatment of lumbar disc herniation by electro-acupuncture | China Journal of Traditional Chinese Medicine and Pharmacy |
| Li 2008 [4]^a^ | The system evaluation of electroacupuncture for lumbar intervertebral disc herniation | Modern Journal of Integrated Traditional Chinese and Western Medicine |
| Jin 2011 [5]^b^ | Shenmai injection for viral myocarditis among children: a Meta-analysis | Changzhou Practical Medical |
| Li 2006 [6]^a^ | Shenmai injection for acute ischemic stroke: a systematic review of randomized controlled trial | Acta Chinese Medicine and Pharmacology |
| Ma 2010 [7]^b^ | Shenmai injection for acute ischemic stroke: a Meta-analysis of randomized controlled trial | Liaoning Journal of Tradtional Chinese Medicine |
| Liu 2012 [8]^a^ | Systematic review on Shenmai Injection treatment of dilated cardiomyopathy | Chinese Traditional Patent Medicine |
| Wang 2011 [9]^a^ | Shenmai Injection in coronary heart disease patients: a systematic review and Meta-analysis | Chinese Journal of Hospital Pharmacy |
| Shi 2012 [10]^a^ | Shenmai Injection for treatment of viral myocarditis: a Meta-analysis | Chinese Traditional Patent Medicine |
| Zhang 2011 [11]^a^ | Systematic reviews and Meta-analysis of Shenmai injection with chemotherapy on treatment of patients with NSCLC | Practical Pharmacy and Clinical Remedies |
| Shu 2008 [12]^a^ | Meta analysis of the treatment for acute cerebral inf arction with Shenmai injection and compound salvia miltiorrhiza injection | Modern Journal of Integrated Traditional Chinese andWestern Medicines |
| Li 2012 [13]^b^ | Shenmai Injection for auxiliary treatment of viral myocarditis: a Meta-analysis | Journal of Chinese Medicinal Materials |
| Zeng 2010 [14]^a^ | Effect of shenmai injection on mortality rate of patients with acute myocardial infarction: a systematic review | Modern Journal of Integrated Traditional Chinese and Western Medicine |
| Cui 2010 [15]^b^ | Shenmai Injection for treatment of coronary heart disease: a Metaanalysis | Lishizhen Medicine and Materia Medica Research |
| She 2003 [16]^b^ | Four kinds of commonly used traditional Chinese medicine injection adverse reaction literature data analysis | Practical Clinical Journal of Integrated Traditional Chinese and Western Medicine |
| Pu 2010 [17]^a^ | Systematic Evaluation on Clinical Therapetic Effect of Acupuncture for Treatment of Gastrointestinal Untoward Reaction by Malignant Tumor Chemotherapy | Lishizhen Medicine and Materia Medica Research |
| Zheng 2014 [18]^a^ | Meta-Analysis on Wrist-Ankle Acupuncture of Cancerous Pain | Journal of Liaoning University of TCM |
| Xinyu 2014 [19]^a^ | Meta-analysis of Banxia Baizhu Tianma decoction in treatment of primary hypertension | Liaoning Journal of Tradtional Chinese Medicine |
| Yixin 2013 [20]^a^ | Meta-analysis and systematic reviews of Liuweidihuang Pills combined with Western medicine on hypertension | Journal of Traditional Chinese Medicine |
| Qainyi 2013 [21]^a^ | Therapeutic effect of Tianma Gouteng decoction combined with captopril in treatment of primary hypertension: a systematic review and meta-analysis | Journal of Anhui University Chinese Medicine |
| Daxing 2011 [22]^a^ | Therapeutic effect of Tianma Gouteng decoction combined with enalapril in treatment of primary hypertension: a systematic review and meta-analysis | Journal of Traditional Chinese Medicine |
| Yi 2006 [23]^a^ | Traditional Chinese medicine for treatment of primary hypertension: a meta-analysis of randomized controlled trials | Shaanxi Journal of Traditional Chinese Medicine |
| Juan 2010 [24]^b^ | Systematic review of traditional Chinese medicine for treatment of primary hypertension | Journal of Traditional Chinese Medicine |
| Hao 2014 [25]^b^ | Efficacy of Yangxue Qingnao granule in treatment of associated symptoms of hypertension: a meta-analysis | Journal of Anhui University Chinese Medicine |
| Dongna 2012 [26]^a^ | Effects of Chinese medicine on elderly isolated systolic hypertension: a meta-analysis | Liaoning Journal of Traditional Chinese Medicine |
| Zheng 2015 [27] | Meta-analysis of children with tic disorder treated with topiramate and haloperidol | Chinese Journal of Child Health Care |
| Xiao 2010 [28]^a^ | Evaluation of clinical randomized control trials of acupuncture for treatment of multiple tics disorders | Lishizhen Medicine and Materia Medica Research |
| Hu 2013 [29]^a^ | Systemic review of Ginkgo biloba extract for vascular dementia | Liaoning Journal of Tradtional Chinese Medicine |
| Wang 2010 [30]^a^ | Meta-analysis of Danhong injection in treating unstable angina pectoris | Chinese Journal of Hospital Pharmacy |
| Xu 2010 [31]^a^ | A systemic review of Danhong injection in treating elderly patients with unstable angina | Liaoning Journal of Traditional Chinese Medicine |
| Xu 2011 [32]^b^ | Meta analysis of Danhong injection in the treatment of unstable angina | Lishizhen Medicine and Materia Medica Research |
| Yang 2011 [33]^a^ | Metaanalysis of Danhong injection in the treatment of unstable angina | China Pharmacy |
| Cui 2012 [34]^b^ | Danhong injection combined conventional western medicine treatmentof unstable angina efficacy and safety evaluation system | Journal of Anhui Traditional Chinese Medical College |

^a^ included in Google Scholar as a citation; ^b^ not found in Google Scholar.

## References

1. X. Qin, P.L., M. Han, Z. Liu, *Systematic review of randomized controlled trials of Xiaoyao powder in treatment of depression.* Journal of Traditional Chinese Medicine, 2010. **6**.

2. al., X.W.e., *Acupuncture for lumbar disc herniation: a systematic review.* Journal of Guangxi Medical University, 2013. **30**(4): p. 562-566.

3. L. Li, H.-S.Z., N.-Y. Gao, B. Chen, and Y.-Y. Shi, *Clinical randomized controlled trials on treatment of lumbar disc herniation by electro-acupuncture.* China Journal of Traditional Chinese Medicine and Pharmacy, 2010. **25**(12): p. 1949-1952.

4. Q. Li, W.P., Y. Mu, Y. Xu, Y. Jiang, K. Ma, *The system evaluation of electroacupuncture for lumbar intervertebral disc herniation.* Modern Journal of Integrated Traditional Chinese and Western Medicine, 2008. **17**(3): p. 325-330.

5. Jin, X.Y., *Shenmai injection for viral myocarditis among children: a Meta-analysis.* Changzhou Practical Medical, 2011. **27**(5): p. 281-285.

6. Li, K.J., *Shenmai injection for acute ischemic stroke: a systematic review of randomized controlled trial.* Acta Chinese Medicine and Pharmacology, 2006. **34**(4): p. 4-7.

7. Li, L.H.M.a.K.J., *Shenmai injection for acute ischemic stroke: a Meta-analysis of randomized controlled trial.* Liaoning Journal of Tradtional Chinese Medicine, 2010. **37**(11): p. 2084-2085.

8. Liu, J.G., *Systematic review on Shenmai Injection treatment of dilated cardiomyopathy.* Chinese Traditional Patent Medicine, 2012. **34**(8): p. 1456-1461.

9. Y. Wang, K.F.M., X. G. Zhang, Y. Z. Hu, and J. Z. Shentu, *Shenmai Injection in coronary heart disease patients: a systematic review and Meta-analysis.* Chinese Journal of Hospital Pharmacy, 2011. **31**(15): p. 1314-1317.

10. Shen, M.S.a.Y., *Shenmai Injection for treatment of viral myocarditis: a Meta-analysis.* Chinese Traditional Patent Medicine, 2012. **34**(10): p. 1882-1886.

11. W. L. Zhang, T.H.Y., B. Liu et al., *Systematic reviews and Meta-analysis of Shenmai injection with chemotherapy on treatment of patients with NSCLC.* Practical Pharmacy and Clinical Remedies, 2011. **14**(2): p. 95-101.

12. Wang, J.Z.S.a.Q.R., *Meta analysis of the treatment for acute cerebral inf arction with Shenmai injection and compound salvia miltiorrhiza injection.* Modern Journal of Integrated Traditional Chinese andWestern Medicines, 2008. **17**(7): p. 973-976.

13. Wu, X.L.a.Y., *Shenmai Injection for auxiliary treatment of viral myocarditis: a Meta-analysis.* Journal of Chinese Medicinal Materials, 2012. **45**(7): p. 1185-1188.

14. Y. J. Zeng, J.W., Y. C. Zhou, and M. X. Huang, *Effect of shenmai injection on mortality rate of patients with acute myocardial infarction: a systematic review.* Modern Journal of Integrated Traditional Chinese and Western Medicine, 2010. **19**(28): p. 3555-3558.

15. W. P. Cui, B.D., R. Qu, G. L. Xu, L. Qin, *Shenmai Injection for treatment of coronary heart disease: a Metaanalysis.* Lishizhen Medicine and Materia Medica Research, 2010. **21**(11): p. 2990-2991.

16. Yang, B.R.S.a.Q.H., *Four kinds of commonly used traditional Chinese medicine injection adverse reaction literature data analysis.* Practical Clinical Journal of Integrated Traditional Chinese and Western Medicine, 2003. **3**(1): p. 53-54.

17. Pu, H.h., Yu, T., Gao, X., Mao, J. J., *Systematic Evaluation on Clinical Therapetic Effect of Acupuncture for Treatmen of Gastrointestinal Untoward Reaction by Malignant Tumor Chemotherapy.* Lishizhen Medicine and Materia Medica Research, 2010. **21**: p. 1476-1480.

18. Zheng, Y., Yu, Y. H. & Fang, F. F., *Meta-Analysis on Wrist-Ankle Acupuncture of Cancerous Pain.* Journal of Liaoning University of TCM, 2014. **16**: p. 152-155.

19. Xinyu C, L.Y., Zixuan L, et al., *Meta-analysis of Banxia Baizhu Tianma decoction in treatment of primary hypertension.* Liaoning J Tradit Chin Med, 2014. **41**: p. 196-9.

20. Yixin G, X.C., Zhenwen Q, et al., *Meta-analysis and systematic reviews of Liuweidihuang Pills combined with Western medicine on hypertension.* J Emerg Tradit Chin Med, 2013. **22**: p. 189-91.

21. Qainyi Z, K.M., Yixin G, et al., *Therapeutic effect of Tianma Gouteng decoction combined with captopril in treatment of primary hypertension: a systematic review and meta-analysis.* J Anhui Univ Chin Med, 2013. **32**: p. 12-7.

22. Daxing D, S.Y., Nan Y, et al., *Therapeutic effect of Tianma Gouteng decoction combined with enalapril in treatment of primary hypertension: a systematic review and meta-analysis.* J Emerg Tradit Chin Med, 2011. **20**(762-4).

23. Yi, R., Aihua, O., Xiaozhong, L., *Traditional Chinese medicine for treatment of primary hypertension: a meta-analysis of randomized controlled trials.* Shaanxi J Tradit Chin Med, 2006. **27**: p. 794-6.

24. Juan, D., *Systematic review of traditional Chinese medicine for treatment of primary hypertension.* J China Tradit Chin Med Inf, 2010. **36**: p. 22-4.

25. Hao, D., Xiaohua, D., *Efficacy of Yangxue Qingnao granule in treatment of associated symptoms of hypertension: a meta-analysis.* J Anhui Univ Chin Med, 2014. **33**: p. 8-11.

26. Dongna L., C.Y., *Effects of Chinese medicine on elderly isolated systolic hypertension: a meta-analysis.* Liaoning J Tradit Chin Med, 2012. **39**: p. 812-5.

27. Zheng, W., Zhang, N., Yang, J.H., Li, X.B., Ma, X., Xiang, Y.Q., *Meta-analysis of children with tic disorder treated with topiramate and haloperidol.* Chin. J. Child Health Care, 2015. **23**: p. 303-306.

28. Xiao, L., Chen, Y.W., Du, Y.H., Gao, X., Lin, X.M., Sun, P., *Evaluation of clinical randomized control trials of acupuncture for treatment of multiple tics disorders.* Lishizhen Med. Materia Medica Res, 2010. **21**: p. 1199-1202.

29. Hu, Q., Tu, X., Zhang, Y., Yang, W. Y., Long, J., *Systemic review of Ginkgo biloba extract for vascular dementia.* Liaoning J. Tradit. Chin. Med., 2013. **4**: p. 683-686.

30. Hu, Z.X.W.a.Y., *Meta-analysis of Danhong injection in treating unstable angina pectoris.* Chinese Journal of Hospital Pharmacy, 2010. **30**(19): p. 1671-1674.

31. G. L. Xu, H.X., H. Y. Cao, L. Qin, *A systemic review of Danhong injection in treating elderly patients with unstable angina.* Liaoning Journal of Traditional Chinese Medicine, 2010. **37**(10): p. 1960-1962.

32. Xu, G.L., Lin, S. M., Xu, H., Qin, L., *Meta analysis of Danhong injection in the treatment of unstable angina.* Lishizhen Medicine and Materia Medica Research, 2011. **22**(3): p. 765-767.

33. Yang, T.F., Zhang, W., Ding, Z. X., Wang, X. X., *Metaanalysis of Danhong injection in the treatment of unstable angina.* China Pharmacy, 2011. **22**(15): p. 1423-1425.

34. R. Cui, K.X., P. Shi, and X. M. Nie, *Danhong injection combined conventional western medicine treatmentof unstable angina efficacy and safety evaluation system.* Journal of Anhui Traditional Chinese Medical College, 2012. **31**(1): p. 14-19.
